# The audiological profile of adults with and without hypertension

**DOI:** 10.6061/clinics/2016(04)02

**Published:** 2016-04

**Authors:** Mariana Aparecida Soares, Seisse Gabriela Gandolfi Sanches, Carla Gentile Matas, Alessandra Giannella Samelli

**Affiliations:** Faculdade de Medicina da Universidade de São Paulo (FMUSP), Departamento de Fisioterapia, Fonoaudiologia e Terapia Ocupacional, Curso de Fonoaudiologia, São Paulo/SP, Brazil

**Keywords:** Hearing loss, Hypertension, Audiometry, Pure Tone, Otoacoustic Emission, Spontaneous

## Abstract

**OBJECTIVE:**

To determine whether there is any influence of systemic arterial hypertension on the peripheral auditory system.

**METHODS:**

This was a cross-sectional study that investigated 40 individuals between 30 and 50 years old, who were divided into groups with and without systemic arterial hypertension, using data from high-frequency audiometry, transient-evoked otoacoustic emissions and distortion-product otoacoustic emissions. The results were compared with those from groups of normal-hearing individuals, with and without systemic arterial hypertension, who underwent the pure-tone audiometry test. All individuals also underwent the following procedures: otoscopy, acoustic immittance measures, pure-tone audiometry at frequencies from 250 to 16000 Hz, transient-evoked otoacoustic emissions test and distortion-product otoacoustic emissions test.

**RESULTS:**

No statistically significant difference was observed between the groups with and without systemic arterial hypertension in either conventional or high-frequency audiometry. Regarding transient-evoked otoacoustic emissions, there was a trend toward statistical significance whereby the systemic arterial hypertension group showed lower results. Regarding distortion-product otoacoustic emissions, the systemic arterial hypertension group showed significantly lower results at the following frequencies: 1501, 2002, and 3003 Hz. A discriminant analysis indicated that the distortion-product otoacoustic emissions variables best distinguished individuals with and without systemic arterial hypertension.

**CONCLUSION:**

Data from this study suggest cochlear dysfunction in individuals with systemic arterial hypertension because their otoacoustic emission results were lower than those in the systemic arterial hypertension group.

## INTRODUCTION

Although systemic arterial hypertension (SAH) is a silent disease, some individuals experience headaches, dizziness, tinnitus, chest pain, and weakness associated with the condition. In addition, SAH may involve secondary hearing changes 1,2.

Diseases that affect the circulatory system can affect the inner ear in many ways 3 as living cells require an adequate supply of oxygen and nutrients to function, and this provision depends on the integrity of the heart and blood vessels 4,5.

It is also believed that presbycusis may be potentiated by hyperviscosity syndrome or microangiopathy caused by diabetes or SAH associated with microcirculatory failure due to vascular occlusion secondary to embolism, hemorrhage, or vasospasm. SAH could thus occur because these factors cause or potentiate hearing loss 6.

Current studies in the literature are controversial regarding whether SAH affects the peripheral auditory system 7. Some studies have revealed a positive association between SAH and hearing loss 5,. However, other studies failed to observe this relationship 11,12. No study has used various methods of peripheral auditory system evaluation simultaneously in individuals with SAH, which demonstrates the importance of this research. The use of other audiological procedures in addition to conventional audiometry can provide earlier and more accurate indications of possible cochlear changes resulting from SAH, which can help preserve the hearing of affected individuals.

The hypothesis of this study is that hypertensive adults with normal hearing, according to conventional audiometry, will exhibit differences in the results of high-frequency audiometry, Transient evoked otoacoustic emissions (TEOAE) and Distortion product otoacoustic emissions (DPOAE) compared with individuals without SAH.

## METHODS

### Subjects

A survey was conducted on the medical records of patients treated at the Hospital Universitário da Universidade de São Paulo-HU-USP from 2009-2012. The following inclusion criteria were used: age between 30 and 50 years; normal hearing detected by conventional audiometry (thresholds ≤25 dBNA); absence of obstruction of the external auditory canal or middle ear disorders; absence of diagnosed metabolic diseases; and no history of noise exposure.

A total of 40 subjects participated:

- Twenty patients, 13 women and seven men aged 31-48 years, (mean±SD 41.4±5.9) with SAH who were diagnosed from one to 23 years previously (mean 7.75). Hypertension was defined as a systolic blood pressure ≥140 mmHg or diastolic blood pressure ≥90 mmHg. The participants were being treated with angiotensin receptor antagonists (nine patients), inhibitors of angiotensin-converting enzymes (five patients), beta-blockers (one patient), calcium channel blockers (one patient), or a thiazide diuretic only (one patient). Three participants were not prescribed any medication for SAH.
- Twenty patients, 13 women and seven men aged 35-50 years, (mean±standard deviation, 45.5±4.7) without SAH.

### Ethical Considerations

The protocol was approved by the Research Ethics Committee of the institution (n1065/10) and informed consent was obtained prior to participation.

### Procedures

The participants underwent an interview to confirm their medical and otological history. The following procedures were then performed:

- Acoustic immittance measurements (tympanometry, acoustic reflex) (AT 235, Interacoustic).
- Pure tone audiometry from 250 to 16,000 Hz (Itera II, Madsen).
- TEOAE (ILO-92, Otodynamics). Cochlear responses were analyzed in the 1,000-5,000 Hz frequency range using a click stimulus at 84 dBpeSPL. Emissions occurred when the following parameters were observed: reproducibility greater than 50%, probe stability greater than 75% and a signal/noise ratio ≥3 dB for four or five of the evaluated frequency bands (1, 2, 3, 4 and 5 kHz).
- DPOAE (ILO-92, Otodynamics). Two different paired frequencies (f1 and f2, being f2/f1 = 1,2) were presented at an intensity of 65dB (f1) and 55dB (f2). Responses occurred when the signal/noise ratio was ≥3 dB relative to the second standard deviation of background noise for five or more of the f2 frequencies (1001, 1501, 2002, 3003, 4004, 5005 and 6006 Hz).

All audiological tests were performed in a soundproof booth.

Statistical analyses were performed using the Hotelling T2 multivariate test to evaluate possible differences between the right and left ears in the measured variables. Thereafter, repeated measures models were adjusted for each of the variables based on a "standard profile", such that the correlation between measurements of the same patient could be incorporated and an expected value was calculated for a particular response. Fisher's exact test and the discriminant analysis technique were also used. P values <0.05 were considered statistically significant.

## RESULTS

### Audiometry

**Figure 1:**
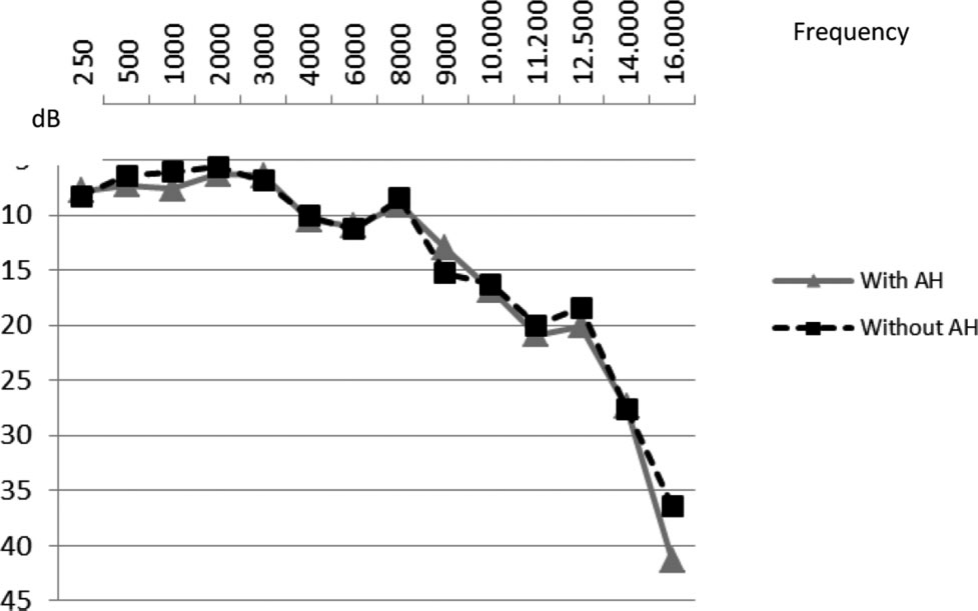
The average of the hearing thresholds of 20 patients with systemic arterial hypertension and 20 subjects without systemic arterial hypertension.

The hearing thresholds of the two groups are shown in Figure 1. There was no statistically significant difference between ears when comparing conventional and high-frequency audiometry (Hotelling's T2 test - *p*-value=0.54). Thus, for this analysis, the standard profile was that of a 43-year-old woman without SAH (Table 1). Regarding age, we noted that there was a statistically significant difference for some frequencies, e.g., increasing age by one year increased the mean threshold. Regarding gender, there was a significant difference only at 4000 Hz, with males exhibiting higher values. There was no statistically significant difference in the between-groups comparison. However, hearing thresholds at 3 kHz in the SAH group were higher than those in the group without SAH.

## TEOAE

There was a statistically significant difference in the comparison between ears (*p*=0.006, Hotelling's T2 test). Therefore, the adopted standard profile referred to the left ear of a 43-year-old woman without SAH (Table 2). Regarding gender, there was a statistically significant difference in the amplitude and reproducibility variables, with men exhibiting lower mean values. Although the SAH group had lower values for TEOAE amplitudes, there was no significant difference between the groups. Regarding the comparison between ears, the right ear showed higher values for most variables.

Regarding the absence of TEOAE, 45% (9 ears) of the ears had absent emissions in the SAH group, whereas 35% of the ears (7 ears) in the group without SAH had absent responses (*p*=0.358 - Fisher's exact test).

## DPOAE

There was no statistically significant difference between the ears (*p*=0.43, Hotelling's T2 test). Thus, the standard profile was a 43-year-old woman without SAH (Table 3). A statistically significant difference in DPOAE amplitudes at 1501, 2002 and 3003 Hz was observed between the groups, with the SAH group exhibiting lower values.

Regarding the absence of DPOAE, we observed that in the SAH group, 5% of the ears (one ear) had absent emissions (at frequencies of 1501, 2002, 3003, 4004, 5005 and 6006 Hz), whereas no ears in the group without SAH had absent responses (*p*=0.246, Fisher's exact test).

### Discriminant analysis

A discriminant analysis was performed to verify whether the analyzed variables could form a linear function capable of classifying patients into groups with and without SAH. This analysis enabled us to investigate which variables could most accurately predict the group to which a particular individual belonged (with or without SAH). Table 4 shows that the highest percentage of the correct score of discriminant analysis is 60% for TEOAE and DPOAE when the classification is made for the two groups simultaneously (e.g., using the results of TEOAE and DPOAE, 60% of the individuals with SAH and 60% of the individuals without SAH were correctly classified within their respective group).

## DISCUSSION

The mean disease duration of the hypertensive group was 7.75 years. Esparza et al. 1 studied a group with four years ago of disease diagnosis (on average). Agarwal et al. 8 evaluated three groups divided into different grades of SAH and the mean disease duration ranged from 3.7 to 9.0 years. The difference in the duration of the SAH observed in these studies may influence the audiological results obtained when we consider the length of time that the microcirculatory impairment was present, which may be a variable that affects the deterioration of the peripheral auditory system to some extent 6.

In the group with SAH, three individuals were not taking any medication for SAH. The presence of associated cardiovascular risk factors, in addition to blood pressure values, should be accounted for when making therapeutic decisions regarding the use of medication. Patients with mild SAH and no other comorbidities may initially be prescribed lifestyle changes 13.

Among the treated patients, only one patient used thiazide diuretics, whereas the others used SAH medication, some of which were associated with diuretics. Of the 16 subjects who used SAH medication, only five were associated with the use of diuretics. Both monotherapy and combination therapy may be effective for controlling blood pressure, and a decision regarding therapy type should be made for each case according to the clinical scenario 14.

Thiazide diuretics are most often used to combat SAH 14. There are studies 15 that emphasize the risk of loop diuretics for hearing, but we did not find any references regarding the ototoxicity of thiazide diuretics in hypertensive adults.

Regarding the use of specific antihypertensive drugs, we found only one case study that correlated the use of beta-blocking drugs with the occurrence of mixed hearing loss 16. Furthermore, only one participant was using a beta-blocker. Esparza et al. 1 stated that the possible deleterious effects of some antihypertensive drugs on cochlear function remain inconclusive because there are no follow-up studies of cochlear function in patients who use this medication.

Regarding audiometry, we observed no statistically significant differences between ears. The literature is consistent regarding the absence of differences between the hearing thresholds in conventional audiometry and those in high-frequency audiometry in the left and right ears 17.

When analyzing the audiometry data, we observed a significant age effect for some frequencies. This effect has been previously described in other studies, which suggest that auditory thresholds worsen with increasing age 18. Regarding the gender variable, the mean hearing threshold value was significantly greater in men at one frequency 17,19. Additionally, for other frequencies evaluated in conventional audiometry and at high frequencies, men had slightly worse hearing thresholds than women (from 3000 Hz). Many studies have reported this difference in hearing thresholds between genders and have mainly attributed this more evident hearing loss in men to lifestyle as men tend to be more exposed to noise 20,21.

We observed no statistically significant differences in audiometry results between the groups. However, starting at a frequency of 3 kHz, the SAH group had worse hearing thresholds.

Many studies have used conventional audiometry to compare individuals with and without SAH. Our results relative to conventional audiometry corroborate the results of Mondelli and Lopes 2 and Wu et al. 22 but disagree with the studies of Esparza et al. 1, Sahin-Yilmaz et al. 23 and Agarwal et al. 8, who observed obvious differences in conventional audiometry thresholds between groups with and without SAH.

Regarding high-frequency audiometry, no studies were observed in the literature that used this method to compare groups with and without SAH. However, we must consider that the number of individuals in the two groups was small and perhaps the small differences in the frequencies above 8 kHz would be more evident with a larger sample size.

Regarding the TEOAE, we observed statistically significant differences in amplitudes between the ears and the responses for the right ear were better in general, which has been previously reported in the literature 24.

In the TEOAE analysis, we observed no significant effect of age, but there was a significant difference for gender, with a lower mean response value for males. Other authors have also reported significant differences in otoacoustic emissions between genders, with a greater amplitude of TEOAE responses in females 24.

When comparing the TEOAE results between the groups, we observed a trend toward statistical significance for the overall reproducibility and amplitude at a frequency of 1 kHz, with lower values in the SAH group. No studies were found in the literature comparing groups with and without SAH using the TEOAE. However, when analyzing the results obtained by audiometry and TEOAE, we can suggest that the second method is more sensitive in detecting possible cochlear changes in hypertensive subjects than the first method. This finding corroborates the literature that indicates that OAE are more sensitive in identifying cochlear changes before they can be observed on an audiogram 15.

The cochlear passive mechanism, described by Békésy, demonstrated the tonotopy of the cochlea. However, it did not explain how this process takes place in live cochlea. The emergence of OAE led to the search for knowledge regarding cochlear active mechanisms. Otoacoustic emissions (TEOAE or DPOAE) occur as a by-product of cochlear mechanisms 15. The recording of OAE enables evaluation of the integrity of outer hair cells, which are considered effectors in the cochlear amplification mechanism due to its biomechanical properties. Its electric mobility enables the inner hair cells, which are essentially sensory, to perform the transduction process. The integrity of the stria vascularis 25 plays an important role in the mechanism of evoked otoacoustic emissions. The stria vascularis has a function in maintaining vital electrochemical gradients for the activity of outer hair cells. Changes in this structure could potentially be related to the reduction response of OAE.

We observed no effect of ear, age, or gender on DPOAE. There were statistically significant differences only in the comparison of DPOAE amplitudes at 1501, 2002 and 3003 Hz between the groups with and without SAH, with lower responses found in the SAH group.

The differences found in DPOAE between the groups with SAH and without SAH are consistent with the study of Sahin-Yilmaz et al. 23, which revealed a smaller number of subjects with DPOAE in the SAH group. In the present study, we found a small difference between the numbers of subjects with DPOAE. However, the smaller DPOAE amplitude in the group with SAH is indicative of cochlear impairment.

Other studies using DPOAE to evaluate individuals with SAH and cardiovascular disease also emphasized the deleterious effect of these disorders on inner ear function 1,7,26.

This procedure has also been used successfully to monitor cochlear function in patients exposed to ototoxic agents 27,28 and we thus suggest that DPOAE should also be used as a complementary evaluation of cochlear function in hypertensive patients.

A qualitative analysis of OAE showed that for TEOAE, more absent responses were obtained in the SAH group (45% *versus* 35% in the group without SAH), and for DPOAE, absent responses occurred only in the group with SAH (5%).

TEOAE are more sensitive to cochlear changes and DPOAE are less sensitive to sub-clinical conditions in adults. Thus, small cochlear changes can affect TEOAE more "quickly" than DPOAE (15), which may explain the higher number of absences in the TEOAE than the DPOAE in both groups. It should also be emphasized that hypertension was not the only variable that may have influenced the OAE results. For instance, because both groups were comprised of 30- to 50-year-old individuals, age may also have acted as an influencing factor on OAE responses because there is a decrease in cochlear function with increasing age.

Therefore, in addition to SAH, such potential covariates (e.g., age, gender) may influence the number of present and absent responses and the amplitude of the OAE responses. Because TEOAE are more sensitive, we hypothesize that the influence of these covariates on this type of emission will soon be observed and is thus unlikely to allow for the precise identification of differences between the groups with and without SAH. Moreover, because DPOAE are less sensitive, even with influences from uncontrolled covariates, DPOAE offer a greater possibility to detect differences that arise from SAH.

The discriminant analysis indicated higher percentages of correct scores for OAE than audiometry, suggesting that OAE was the audiological evaluation that allowed the differentiation of individuals with and without SAH.

Furthermore, based on the results of the discriminant analysis combined with those of the quantitative analyses, we can suggest that DPOAE in general is the best method for classifying individuals within groups with or without SAH.

Although the TEOAE and DPOAE techniques are complementary, DPOAE is more suitable for advanced clinical investigation in adult patients because it is more flexible and allows for a more precise analysis than TEOAE 15. According to our results, DPOAE is also indicated in the evaluation of hypertensive adults.

It is important to mention that the present study was based on a small sample. We therefore suggest that future studies should be conducted with a larger number of hypertensive individuals. Furthermore, longitudinal studies of hearing in individuals with this condition may contribute significantly to the identification of the effect of SAH on hearing.

Because the individuals who participated in this research had hearing thresholds within normal conventional audiometry limits, had no history of exposure to noise or other metabolic diseases and had their statistical analyses adjusted for possible influences arising from gender or age, we consider that the differences observed between the procedures (high-frequency audiometry, TEOAE and DPOAE) between hypertensive and non-hypertensive patients, regardless of significance, could be related to the presence or absence of this condition, which can cause bleeding in the inner ear 4,29,30 and microcirculatory failure 6.

Thus, although controversy remains regarding the influence of SAH on hearing, it is necessary to closely investigate hypertensive patients, who must undergo audiological monitoring that includes not only conventional but also high-frequency audiometry and/or otoacoustic emission testing that can identify possible cochlear malfunctions earlier.

The differences observed between hypertensive and non-hypertensive patients using various procedures allowed us to suggest that hypertensive individuals have cochlear dysfunction that is not detected by conventional audiometry. Furthermore, the most sensitive tool for discrimination between individuals with and without SAH was DPOAE, rather than high-frequency audiometry and TEOAE.

## AUTHOR CONTRIBUTIONS

Soares MA and Samelli AG were in charge of data collection. Soares MA, Sanches SG, Matas CG and Samelli AG collaborated in the data analysis. Samelli AG was responsible for the general supervision of the execution stages. All the authors drafted the manuscript.

## Figures and Tables

**Table 1:** Estimates and *p*-values of the model effects of the audiometry variables.

**dBNA estimate (***p***-value)**				
Variable	Estimate for standard profile	Estimate for age (each year)	Estimate for male gender	Estimate for SAH group
**250 Hz**	8.05 (<0.001)*	-0.12 (0.235)	-0.18 (0.876)	0.01 (0.99)
**500 Hz**	7.13 (<0.001)*	-0.05 (0.662)	-2.49 (0.084)**	1.11 (0.446)
**1000 Hz**	7.09 (<0.001)*	0.07 (0.584)	-2.36 (0.092)	1.22 (0.389)
**2000 Hz**	6.01 (<0.001)*	-0.06 (0.636)	-1.42 (0.283)	0.86 (0.545)
**3000 Hz**	6.17 (<0.001)*	0.07 (0.628)	2.42 (0.126)	-0.78 (0.624)
**4000 Hz**	9.18 (<0.001)*	0.08 (0.577)	3.16 (0.05)*	-0.08 (0.961)
**6000 Hz**	11.45 (<0.001)*	0.21 (0.172)	0.67 (0.691)	-1.13 (0.516)
**8000 Hz**	8.63 (<0.001)*	0.04 (0.794)	-0.11 (0.954)	-0.44 (0.825)
**9000 Hz**	14.22 (<0.001)*	0.01 (0.969)	2.99 (0.213)	-2.28 (0.352)
**10.000 Hz**	15.25 (<0.001)*	0.19 (0.455)	3.95 (0.156)	-0.27 (0.922)
**11.200 Hz**	19.92 (<0.001)*	0.60 (0.059)**	3.80 (0.271)	-1.61 (0.645)
**12.500 Hz**	18.81 (<0.001)*	1.08 (0.009)*	5.19 (0.238)	-2.74 (0.539)
**14.000 Hz**	29.87 (<0.001)*	1.86 (< 0.001)*	4.60 (0.365)	-7.95 (0.130)
**16.000 Hz**	39.70 (<0.001)*	1.94 (< 0.001)*	2.00 (0.641)	-3.17 (0.472)

* = statistically significant

** = marginal *p*-value (tendency toward statistical significance).

**Table 2:** Estimates and *p*-values of the adjusted repeated measures model effects of the transient-evoked otoacoustic emission variables.

**dB Estimate (***p***-value)**					
Variable	Estimate for standard profile	Estimate for age (each year)	Estimate for male gender	Estimate for SAH group	Estimate for right ear
**Amplitude (Response)**	13.82 (<0.001)*	0.13 (0.254)	-2.73 (0.031)*	-1.81 (0.154)	1.60 (0.003)
**Reproducibility**	89.65 (<0.001)*	0.19 (0.453)	-6.25 (0.031)*	-5.17 (0.078)**	2.72 (0.067)**
**1 kHz (%)**	85.20 (<0.001)*	-0.18 (0.713)	5.42 (0.323)	-7.64 (0.177)	4.55 (0.001)*
**2 kHz (%)**	90.85 (<0.001)*	0.23 (0.488)	-4.67 (0.203)	-3.37 (0.367)	3.77 (0.200)
**3 kHz (%)**	83.95 (<0.001)*	0.35 (0.636)	-18.33 (0.028)*	2.93 (0.723)	-3.17 (0.423)
**4 kHz (%)**	73.95 (<0.001)*	-0.43 (0.556)	-25.97 (0.003)*	0.83 (0.921)	5.32 (0.175)
**5 kHz (%)**	43.90 (<0.001)*	-0.53 (0.515)	-19.81 (0.031)*	3.26 (0.721)	12.57(0.067)**
**1 kHz (dB)**	9.71 (<0.001)*	0.03 (0.843)	1.30 (0.427)	-2.82 (0.097)**	2.30 (0.001)*
**2 kHz (dB)**	12.67 (<0.001)*	0.23 (0.092)**	-2.25 (0.135)	-2.31 (0.135)	1.95 (0.037)*
**3 kHz (dB)**	10.32 (<0.001)*	0.22 (0.164)	-3.85 (0.028)*	-1.15 (0.509)	-0.60 (0.441)
**4 kHz (dB)**	7.68 (<0.001)*	-0.08 (0.577)	-5.30 (0.001)*	-1.25 (0.430)	0.52 (0.491)
**5 kHz (dB)**	1.96 (0.061)**	-0.08 (0.455)	-2.11 (0.072)**	0.30 (0.800)	1.17 (0.237)

* = statistically significant difference;

** = marginal *p*-value (tendency toward statistical significance).

**Table 3:** Estimates and *p*-values of the adjusted repeated measures model effects of DPOAE variables.

dB Estimate (*p*-value)				
Variable	Estimate for standard profile	Estimate for age (each year)	Estimate for male gender	Estimate for SAH group
**1001 Hz**	15.31 (<0.001)*	0.02 (0.913)	2.78 (0.192)	-1.73 (0.425)
**1501 Hz**	22.74 (<0.001)*	0.07 (0.672)	2.10 (0.290)	-6.49 (0.003)*
**2002 Hz**	20.04 (<0.001)*	0.04 (0.829)	0.05 (0.982)	-5.04 (0.040)*
**3003 Hz**	19.03 (<0.001)*	0.10 (0.527)	-1.66 (0.341)	-3.90 (0.033)*
**4004 Hz**	20.16 (<0.001)*	-0.15 (0.228)	-1.75 (0.212)	-1.31 (0.360)
**5005 Hz**	21.51 (<0.001)*	-0.23 (0.174)	-1.28 (0.484)	-1.74 (0.358)
**6006 Hz**	14.50 (<0.001)*	-0.15 (0.512)	-2.26 (0.362)	-1.71 (0.498)

* = statistically significant difference;

** = marginal *p*-value (tendency toward statistical significance).

**Table 4:** The percentage of correct scores in the discriminant analysis for both ears combined.

	Correct score		
Variables	Without SAH	With SAH	Total
**Audiometry**	47.5%	42.5%	45.0%
**TEOAE (Amplitude and Repro)**	52.5%	65.0%	58.7%
**TEOAE (Other variables)**	62.5%	57.5%	60.0%
**DPOAE**	65.0%	55.0%	60.0%
